# Preoperative prostate health index predicts adverse pathology and Gleason score upgrading after radical prostatectomy for prostate cancer

**DOI:** 10.1186/s12894-020-00711-5

**Published:** 2020-09-07

**Authors:** Vojtech Novak, Stepan Vesely, Hana Luksanová, Richard Prusa, Otakar Capoun, Vojtech Fiala, Olga Dolejsová, Hana Sedlacková, Radek Kucera, Jiri Stejskal, Miroslav Zalesky, Marko Babjuk

**Affiliations:** 1 Department of Urology, Charles University 2nd Faculty of Medicine University Hospital Motol, Prague, Czech Republic; 2 Department of Medical Chemistry and Clinical Biochemistry, Charles University 2nd Faculty of Medicine University Hospital Motol, Prague, Czech Republic; 3 Department of Urology, General University Hospital and 1st Faculty of Medicine, Charles University, Prague, Czech Republic; 4 Department of Urology, University Hospital and Faculty of Medicine in Pilsen, Pilsen, Czech Republic; 5 Department of Immunochemistry, University Hospital and Faculty of Medicine in Pilsen, Pilsen, Czech Republic; 6 Department of Urology and 1st and 3rd Medical Faculty, Thomayer Hospital, Charles University, Prague, Czech Republic; 7 Medical University of Vienna, Vienna, Austria

**Keywords:** Prostate cancer, Radical prostatectomy, PSA isoforms, Prostate health index, Adverse pathology

## Abstract

**Background:**

We aimed to explore the utility of prostate specific antigen (PSA) isoform [− 2] proPSA and its derivatives for prediction of pathological outcome after radical prostatectomy (RP).

**Methods:**

Preoperative blood samples were prospectively and consecutivelyanalyzed from 472 patients treated with RP for clinically localized prostate cancerat four medical centers. Measured parameters were PSA, free PSA (fPSA), fPSA/PSA ratio, [− 2] proPSA (p2PSA), p2PSA/fPSA ratio and Prostate Health Index (PHI)(p2PSA/fPSA)*√PSA]. Logistic regression models were fitted to determine the accuracy of markers for prediction of pathological Gleason score (GS) ≥7, Gleason score upgrading, extracapsular extension of the tumor (pT3) and the presence of positive surgical margin (PSM). The accuracy of predictive models was compared using area under the receiver operating curve (AUC).

**Results:**

Of 472 patients undergoing RP, 339 (72%) were found to have pathologic GS ≥ 7, out of them 178 (53%) experienced an upgrade from their preoperative *GS* = 6. The findings of pT3 and PSM were present in 132 (28%) and 133 (28%) cases, respectively. At univariable analysis of all the preoperative parameters, PHI was the most accurate predictor of pathological GS ≥7 (OR 1.02, 95% CI 1.01–1.03, *p*<0.001), GS upgrading (OR 1.02, 95% CI 1.01–1.03, *p*<0.003), pT3 disease (OR 1.01, 95% CI 1.00–1.02, *p*<0.007) and the presence of PSM (OR 1.01, 95% CI 1.00–1.02, *p*<0.002). Adding of PHI into the base multivariable model increased significantly the accuracy for prediction of pathological GS by 4.4% to *AUC* = 66.6 (*p* = 0.015) and GS upgrading by 5.0% to *AUC* = 65.9 (*p* = 0.025), respectively.

**Conclusions:**

Preoperative PHI levels may contribute significantly to prediction of prostate cancer aggressiveness and expansion of the tumor detected at final pathology.

## Background

Prostate cancer (PC) is the second most common cancer to affect men worldwide with rising incidence over the last 15 years [1]. Wide clinical implementation of serum prostate specific antigen (PSA) test has led to improvement of early PC detection and reduction in disease specific mortality [2]. However, the diagnostic accuracy of PSA is limited with frequent overdetection of clinically insignificant cancer. Moreover, up to 30% of patients with low-risk PC at biopsy are found to have features of moreaggressive disease at radical prostatectomy (RP) [3]. Multivariable models prior to prostate biopsy or RP lack the accuracy in predicting of cancer aggressiveness at final pathology. In order to classify correctly those patients with clinically significant prostate cancer, the need for novel biomarkers, especially in combination with approved clinical risk factors, is extensively debated.

Several promising blood- and urine- based biomarkers have been suggested for clinical use, with varying results. Among these is [− 2]proPSA (p2PSA) derivative, namely the Prostate Health Index (PHI), which has demonstrated improved performance characteristics to PSA [4–6]. Although many studies have evaluated the relationship between the level of PHI and the result of biopsy Gleason score (GS), little is known about the utility of preoperative PHI for prediction of adverse pathological features in RP specimen. In a single center study, Guazzoni et al. found that p2PSA and PHI are strong predictors of PC characteristics at final pathology after the surgery [7]. This finding was later confirmed in a multicenter study; however, decision curve analysis did not prove greater clinical net benefit of prediction models incorporating PHI [8]. A recent single center study in 437 patients suggests PHI to be not only good diagnostic tool, but an independent predictor of biochemical recurrence after RP [9].

Thus, there is insufficient evidence to demonstrate the relationship of PSA isoforms with tumor aggressiveness and further studies are necessary to validate these tests in order to aid in personalized treatment decisions. The aim of this study was to determine the accuracy of p2PSA and its derivatives in predicting pathology outcome within prospectively collected multicenter cohort of patients who underwent RP for clinically localized PC.

## Methods

### Study population

The study cohort consisted of 472 patients with transrectal biopsy- proven clinically localized PC, who underwent either open or laparoscopic RP at four urologic centres in Czech Republic between 2014 and 2018 (University Hospital Motol, Prague; General University Hospital, Prague; Thomayer University Hospital, Prague; University Hospital Pilsen, Pilsen). The study protocol was approved by the institutional review board at each site before initiation, and the study was performed in compliance with their respective requirements.

### Methods

All patients included in our study had complete clinical and pathologic data. Clinical data consisted of age at surgery, PSA, free PSA (fPSA), p2PSA, %p2PSA (p2PSA/fPSA), PHI (p2PSA/fPSA)*√PSA], digital rectal examination (DRE), biopsy GS, number of biopsy cores and number of positive biopsy cores. Pathological data consisted of pathologic stage, pathological GS and surgical margin status. Patients with incomplete clinical data (*n* = 68) were not included to the final analysis. Statistical comparison of clinico-pathological characteristics of patients excluded from the study did not differ significantly from the analysed cohort (Chi-square test, Mann-Whitney test). The specimens were processed and evaluated by experienced pathologists who were blind to the blood tests results. Prostate cancer was graded according to the 2005 consensus conference of International Society of Urological Pathology definitions [10]. Clinically significant prostate cancer was defined as Gleason score 7 or greater. Surgical specimens were processed and evaluated according to the Stanford Protocol, and prostate cancer was staged according to the 2002 TNM staging system [11]. A positive surgical margin was defined as the presence of tumour at the inked surface of the resected specimen.

Patients with acute prostatitis, urinary tract infection, prior transurethral resection of the prostate, recent prostatic manipulation or medications that might alter serum PSA concentrations (for example finasteride or dutasteride) were excluded from study. Subjects received no prior treatment for PC at the time of blood draw. A preoperative blood sample was collected before any manipulations that might cause a transient increase in the levels of biomarker. All blood samples were processed with the UniCel Dx1018 Immunoassay System analyser (Beckman Coulter Inc., Brea, CA, USA) and were managed according to the criteria described by Semjonow et al. [12]. Blood sample analysis with determination of PSA, fPSA, free to PSA ratio (%fPSA) and PHI was performed using the Hybritech calibration Beckman Coulter in all patients.

### Statistical analysis

The Mann-Whitney test and chi-square test were used to compare continuous variables and proportions in categorical variables between groups, respectively. Univariable and multivariable logistic regression models were fitted to determine the ability of various preoperative variables to predict unfavorable pathological outcome. Receiver operating characteristic (ROC) curve was created for each variable. Predictive accuracy of each variable was quantified as the area under the receiver operating characteristics curve (AUC). A multivariable logistic model assessed their predictive independency. The significance of the difference between AUCs of different predictive models was assessed with the method of DeLong et al. [13]. All statistical analyses were conducted with R - statistical package version 3.5.1. (R Core Team, Vienna, Austria, 2018).

## Results

**Fig. 1:**
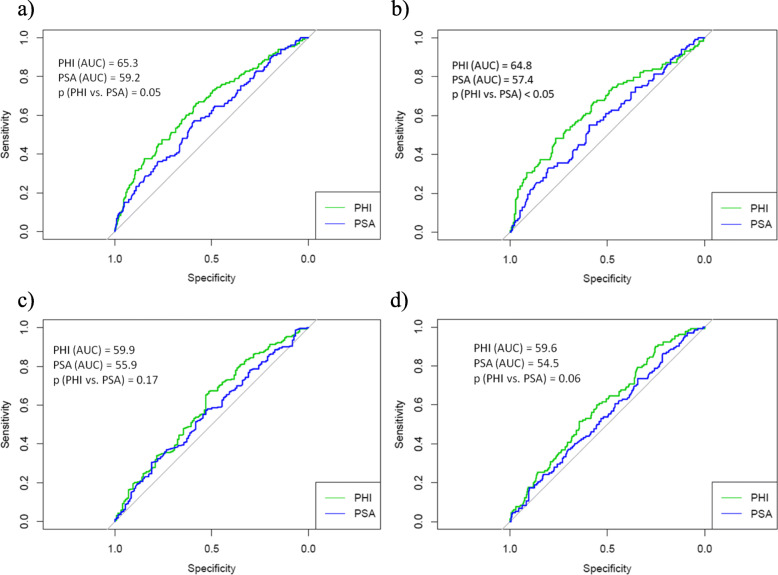
Receiver operating characteristics curves and calculated area under the curve (AUC) values depicting the accuracy of PSA and PHI in prediction of **a**) pathological Gleason score ≥ 7, **b**) Gleason score upgrading, **c**) pathological pT3 disease and **d**) the presence of positive surgical margin (PSM)

Characteristics of the study population are displayed within Table 1. Patients with significant PC at final pathology had higher preoperative PHI level (65.89 ± 33.26 vs 52.23 ± 25.11; *p* < 0.001), higher preoperative PSA level (10.32 ± 7.66 vs 8.19 ± 4.51; *p* = 0.002) and higher frequency positive DRE (33.1% vs 23.3%; *p*<0.05) in the comparison with the rest of patients. At the biopsy, the proportion of patients with GS ≥7 was 37.3% while at final pathology, the proportion raised to 71.8%. Out of 296 patients with biopsy GS 6, a total of 178 (60%) progressed to significant PC at final histology. Patients with upgrading to significant PC at final histology had higher preoperative PHI level than the patients who had GS 6 both at biopsy and final pathology (62.24 ± 25.71 vs 52.67 ± 25.71; *p* < 0.001). As shown in Fig. 1, PHI was significantly stronger predictor of pathological GS ≥ 7 and GS upgrading in the comparison with conventional PSA (*AUC* = 65.3 vs *AUC* = 59.2; *p* = 0.05 and *AUC* = 64.8 vs *AUC* = 57.4; *p* < 0.05, respectively). On multivariable analyses, PHI proved to be independent predictor of GS ≥ 7 and GS upgrading (Tables 2 and 3). Base multivariable model (age, PSA, fPSA and clinical stage) reached the predictive accuracy of *AUC* = 62.2 for pathological GS ≥ 7 and *AUC* = 60.9 for GS upgrading. Adding of PHI into the base multivariable model increased significantly the predictive accuracy from *AUC* = 62.2 to *AUC* = 66.6 (*p* < 0.015) for GS ≥ 7 and from *AUC* = 60.9 to *AUC* = 65.9 (*p* < 0.025) for GS upgrading. No improvement of the base multivariable model appeared after an addition of p2PSA (for GS ≥ 7 *AUC* = 62.1; *p* = 0.585 and for GS upgrading *AUC* = 60.8; *p* = 0.921) and %p2PSA (for GS ≥ 7 *AUC* = 64.9; *p* = 0.101 and for GS upgrading *AUC* = 64.4; *p* = 0.116).

**Table 1:** Clinicopathologic characteristics of 472 patients undergoing radical prostatectomy for clinically localized prostate cancer at four medical centers

Variable		Pathological Gleason score ≥ 7	Pathological Gleason score = 6	*p* value
Number of patients	472	339	133	
age (years, median, range)	65.4 (60.28–69)	65.7 (61.23–69.2)	65.2 (60.03–67.4)	0.123
PSA (ng/ml, median, range)	8.04 (5.78–11.73)	8.98 (6.53–12.06)	7.02 (5.03–9.21)	0.002
fPSA (ng/ml, median, range)	0.91 (0.61–1.35)	0.82 (0.52–1.08)	1.02 (0.87–1.41)	0.800
%fPSA (ratio, median, range)	9.29 (4.76–13.35)	7.23 (4.22–11.8)	11.13 (8.97–14.63)	0.023
p2PSA (pg/ml, median, range)	17 (12–26)	19 (14–29)	14 (10–22)	0.058
%p2PSA (ratio, median, range)	19.60 (15.33–25.79)	20.32 (19.12–26.87)	17.8 (13.91–21.82)	0.007
PHI (median, range)	55.68 (42.24–72.82)	59.53 (51.11–74.61)	45.23 (38.07–59.22)	< 0.001
DRE; n (%)				
negative	329 (69.7)	227 (67.0)	102 (76.7)	
positive	143 (30.3)	112 (33.0)	31 (23.3)	< 0.005
Biopsy Gleason score; n (%)				
6	296 (62.7)	179 (52.8)	117 (88.0)	
7	141 (29.9)	128 (37.8)	13 (9.8)	
> 7	35 (7.4)	32 (9.4)	3 (2.2)	0.032
Pathological tumor stage; n (%)				
pT2	340 (72.1)	226 (66.7)	114 (85.7)	
pT3	132 (27.9)	113 (33.3)	19 (14.3)	< 0.003
Surgical margin status; n (%)				
R0	339 (71.8)	230 (67.8)	109 (82.0)	
R1	133 (28.2)	109 (32.2)	24 (18.0)	< 0.050

*PSA* prostate specific antigen - PSA, *fPSA* free PSA, *%fPSA* fPSA/PSA, *%p2PSA* /fPSA ratio, *PHI* Prostate health index, *DRE* digital rectal examination, *R0* negative surgical margin, *R1* positive surgical margin

**Table 2:** Univariable and multivariable logistic regression model of preoperative variables predicting pathological Gleason score ≥ 7

Variables	Univariable analysis		Multivariable analysis			
	OR (95% CI)	*p* value	Base model OR (95% CI)	*p* value	Base model + PHI OR (95% CI)	*p* value
Age	1.02 (0.99–1.06)	0.1462	1.02 (0.99–1.05)	0.1660	1.02 (0.99–1.06)	0.1437
DRE	1.62 (1.01–2.60)	0.0395	1.71 (1.06–2.76)	0.0261	1.63 (1.00–2.65)	0.0440
PSA	1.07 (1.02–1.12)	0.0025	1.08 (1.03–1.14)	0.0013	1.05 (0.99–1.09)	0.0531
fPSA	0.98 (0.94–1.03)	0.4477	0.92 (0.82–1.02)	0.1146	0.96 (0.89–1.04)	0.2925
p2PSA	1.01 (0.99–1.02)	0.1212				
%p2PSA	1.03 (1.01–1.06)	0.0155				
PHI	1.02 (1.01–1.03)	< 0.0001			1.02 (0197–1.03)	0.0015

*DRE* digital rectal examination, *PSA* prostate specific antigen, *fPSA* free PSA, *p2PSA* [−2]proPSA, *%p2PSA* 2pSA/fPSA ratio, *PHI* Prostate Health Index, *OR* Odds ratio, *CI* confidence interval

**Table 3:** Univariable and multivariable logistic regression model of preoperative variables predicting Gleason sum upgrading

Variables	Univariable analysis		Multivariable analysis			
	OR (95% CI)	*p* value	Base model OR (95% CI)	*p* value	Base model + PHI OR (95% CI)	*p* value
Age	1.02 (0.98–1.05)	0.2943	1.02 (0.98–1.05)	0.3404	1.02 (0.98–1.06)	0.3068
DRE	1.51 (0.87–2.59)	0.1325	1.61 (0.93–2.81)	0.0855	1.65 (0.94–2.89)	0.0755
PSA	1.06 (1.00–1.11)	0.0311	1.07 (1.01–1.13)	0.0150	1.04 (0.98–1.10)	0.1872
fPSA	0.99 (0.83–1.19)	0.9579	0.88 (0.69–1.12)	0.2901	0.93 (0.73–1.16)	0.4979
p2PSA	0.99 (0.99–1.01)	0.7888				
%p2PSA	1.03 (0.99–1.06)	0.0666				
PHI	1.02 (1.01–1.03)	0.0025			1.02 (1.00–1.03)	0.0153

*DRE* digital rectal examination, *PSA* prostate specific antigen, *fPSA* free PSA, *p2PSA* [− 2]proPSA, *%p2PSA* p2PSA/fPSA ratio, *PHI* Prostate Health Index, *OR* Odds ratio, *CI* confidence interval

Patients with pT3 and positive surgical margin (PSM) at final pathology had significantly higher preoperative PHI level in the comparison with the rest of patients (70.82 ± 38.58 vs 58.89 ± 28.32; *p* < 0.001 and 70.02 ± 39.28 vs 58.89 ± 27.82; *p* < 0.001, respectively). Prostate Health Index proved to be independent predictor of pT3 or PSM in multivariable settings (Tables 4 and 5). Base multivariable model (age, PSA, fPSA, clinical stage and biopsy GS) reached the predictive accuracy of *AUC* = 58.8 for pT3 disease and *AUC* = 58.3 for PSM. Adding of PHI into the base multivariable model increased the predictive accuracy from *AUC* = 58.8 to *AUC* = 61.2 (*p* < 0.182) for pT3 disease and from *AUC* = 58.3 to *AUC* = 61.2 (*p* < 0.187) for the presence of PSM, however in both cases the increase was not significant. Similarly, no improvement of the base multivariable model appeared after an addition of p2PSA (for pT3 *AUC* = 60.5; *p* = 0.445 and for PSM *AUC* = 61.0; *p* = 0.283) and %p2PSA (for pT3 *AUC* = 60.4; *p* = 0.436 and for PSM *AUC* = 60.8; *p* = 0.272).

**Table 4:** Univariable and multivariable logistic regression model of preoperative variables predicting pathological T3 disease

Variables	Univariable analysis		Multivariable analysis			
	OR (95% CI)	*p* value	Base model OR (95% CI)	*p* value	Base model + PHI OR (95% CI)	*p* value
Age	0.99 (0.96–1.02)	0.6902	0.99 (0.96–1.03)	0.8237	0.99 (0.94–1.03)	0.8436
DRE	1.64 (1.06–2.54)	0.0233	1.68 (1.07–2.61)	0.0202	1.59 (1.01–2.49)	0.0404
Biopsy GS	1.79 (1.07–2.98)	0.0043	1.78 (1.08–2.85)	0.0135	1.65 (1.02–2.38)	0.0324
PSA	1.04 (1.01–1.07)	0.0124	1.04 (1.00–1.07)	0.0323	1.02 (0.98–1.05)	0.3022
fPSA	1.01 (0.97–1.05)	0.5891	0.99 (0.93–1.05)	0.7388	1.00 (0.95–1.06)	0.8705
p2PSA	1.02 (1.01–1.03)	0.0001				
%p2PSA	1.02 (1.01–1.05)	0.0243				
PHI	1.01 (1.00–1.02)	0.0007			1.01 (1.00–1.02)	0.0112

*DRE* digital rectal examination, *Biopsy GS* Biopsy Gleason score, *PSA* prostate specific antigen, *fPSA* free prostate specific antigen, *p2PSA* [− 2]proPSA, *%p2PSA* [− 2]proPSA/fPSA ratio, *PHI* Prostate health index, *OR* Odds ratio, *CI* confidence interval

**Table 5:** Univariable and multivariable logistic regression model of preoperative variables predicting the presence of positive surgical margin at final pathology

Variables	Univariable analysis		Multivariable analysis			
	OR (95% CI)	*p* value	Base model OR (95% CI)	*p* value	Base model + PHI OR (95% CI)	*p* value
Age	0.99 (0.97–1.03)	0.9808	1.00 (0.97–1.03)	0.9743	1.00 (0.97–1.04)	0.9514
DRE	1.44 (1.06–2.86)	0.0067	1.45 (1.07–2.65)	0.0066	1.43 (1.02–2.58)	0.0121
Biopsy GS	1.52 (1.04–2.88)	0.0434	1.51 (1.05–2.87)	0.0355	1.48 (1.05–2.36)	0.0462
PSA	1.03 (0.99–1.06)	0.0970	1.02 (0.98–1.06)	0.2120	0.99 (0.96–1.04)	0.8618
fPSA	0.98 (0.91–1.06)	0.6749	0.97 (0.84–1.11)	0.6051	0.98 (0.88–1.09)	0.6931
p2PSA	1.02 (1.01–1.03)	0.0018				
%p2PSA	1.03 (1.01–1.06)	0.0036				
PHI	1.01 (1.00–1.02)	0.0014			1.01 (1.00–1.02)	0.0066

*DRE* digital rectal examination, *Biopsy GS* Biopsy Gleason score, *PSA* prostate specific antigen, *fPSA* free prostate specific antigen, *p2PSA* [− 2]proPSA, *%p2PSA* [− 2]proPSA/fPSA ratio, *PHI* Prostate health index, *OR* Odds ratio, *CI* confidence interval

## Discussion

Prostate cancer has been known as the second most common cancer types in current male population, and the one of the leading cause of death for cancer in males [1, 14]. Due to the widespread use of PSA as a primary screening tool for PC, the proportion of low-risk PC has increased considerably, and treatment options for PC are known to differ by risk stratification. Patients with localized PC are advised to undergo not only invasive procedures involving surgery or radiotherapy, but also the modality of active surveillance. Nevertheless, it has been published that up to 30% of patients with low-risk PC at biopsy are found to have features of aggressive disease at RP [3]. In our series of 472men with PC, the proportion of patients with GS ≥ 7 at biopsy was 37.3% while at final pathology, the proportion raised to 71.8%. PHI included into multivariable model proved to be the strongest independent predictor of both pathological GS and GS upgrading.

In recent years, the most common strategy for assessing risk of aggressive PC at final pathology was the preoperative prediction tool combining established parameters like PSA, biopsy GS and clinical stage. Many researchers have investigated new predictors of PC aggressiveness including novel biomarkers [15]. Although some new genomic tests have been recently introduced, none of these costly tools is used in a clinical routine setting. Several authors have analyzed the relationship between PSA isoforms and final pathology in patients treated with RP for clinically localized PC [16]. Guazzoni in the analysis of 350 consecutive men found PHI to be significant predictor of GS ≥ 7 (*AUC* = 74) and/or pT3 disease (*AUC* = 72) [7]. These results were confirmed in a multicenter study performed in 489 patients; however, decision curve analysis did not prove greater clinical net benefit of prediction models incorporating PHI [8]. A recent single center study in 437 patients described lower predictive power of PHI in prediction of GS ≥ 7 (*AUC* = 65) and/or pT3 disease (*AUC* = 70), however the authors suggests PHI to be independent predictor of biochemical recurrence after radical prostatectomy (*AUC* = 62) [9]. Our results confirm the ability of PHI to predict GS ≥ 7 (*AUC* = 65) and/or pT3 disease (*AUC* = 60). Moreover, PHI outperformed other preoperative variables in predicting the presence of PSM. Multivariable models showed improvement in the predictive accuracy after inclusion of PHI, although the gain did not reach the level of statistical significance in prediction of pT3 disease and the presence of PSM.

Positive surgical margin is defined as the histological presence of cancer cells at the inked margin on the RP specimen and a number of studies have demonstrated an association between PSM and a risk of disease progression [17, 18]. A tumor marker would be expected to correlate with the grade of differentiation or tumor extent but not necessarily with margin status, which is most likely to be confounded by surgical technique and pathological interpretation. To our knowledge, there is no published data about the PSA isoforms in the relation to surgical margin status after RP. However, in our series of 472 patients after RP, approximately 28% of patients reported PSM and PHI was the strongest predictor of PSM (*AUC* = 60%) among all the preoperative variables.

Our study has some inherent limitations. The level of accuracy promoted by PHI in our study was noticeably lower in the comparison with previously reported data. The difference may be explained by the multi-institutional nature of the study, when indication criteria for prostatectomy and patients engagement in active surveillance may differ among four institutions involved. In the current study the proportion of low-risk patients with biopsy *GS* = 6 was considerably higher (63%) in the comparison with above cited papers (36–53%), which supports our explanation [8, 9]. Even though all blood samples were managed in the same standardized way at each contributing institution, the data might be influenced by preanalytical and analytical bias. The relatively short follow-up after the RP prevented us from investigating the association of preoperative PHI and the risk of disease progression.

## Conclusions

Results from the current study show that PHI could be useful predictor of PC aggressiveness and expansion of the tumor after radical prostatectomy. Moreover, higher value of PHI was associated with the risk of GS upgrading. However, further research is warranted to confirm our findings through the use of multivariable predictive nomograms incorporating PHI.

## Data Availability

The datasets used and analysed during the current study are available from the corresponding author on reasonable request.

## Abbreviations

AUC: Area under the receiver operating characteristics curve
confidence interval: CI
pT3: Extracapsular extension of the tumor
fPSA: Free PSA
GS: Gleason score
Hazard ratio: HR
PC: Prostate cancer
PHI: Prostate Health Index
PSA: Prostate specific antigen
PSM: Positive surgical margin
RP: Radical prostatectomy
ROC: Receiver operating characteristic
negative surgical margin: R0
positive surgical margin: R1
p2PSA: [− 2] proPSA
